# Progressive Relaxation Behavior and Relaxation Dynamics of sPS Gels upon Controlled Heating

**DOI:** 10.3390/polym10050526

**Published:** 2018-05-14

**Authors:** Yanzhi Zhao, Juying Zhou, Yanjiao Lan, Pengfei Li, Fangkai Du, Fuhou Lei, Hao Li, Qin Huang

**Affiliations:** 1 School of Chemistry and Chemical Engineering, Guangxi University for Nationalities, Nanning 530006, China; zhaoyzsense@163.com (Y.Z.); lyj9395@163.com (Y.L.); lipfgxun@126.com (P.L.); dufangkai501@163.com (F.D.); leifuhou@gxun.cn (F.L.); lihaospace@163.com (H.L.); 2 Guangxi Key Laboratory of Chemistry and Engineering of Forest Products, School of Chemistry and Chemical Engineering, Guangxi University for Nationalities, Nanning 530006, China

**Keywords:** intrinsic fluorescence, fluorescence anisotropy, syndiotactic polystyrene gel, relaxation behavior, melting

## Abstract

Progressive relaxation behavior of syndiotactic polystyrene (sPS) chains in sPS gel was detected in the course of melting via the application of intrinsic fluorescence and fluorescence anisotropy techniques. The melting process included a dissociative process of the network at lower temperature and a relaxation process from helix to worm-like chains at higher temperature. The dynamics of structural relaxation behavior was discovered by intrinsic fluorescence technique, and an abrupt bend emerged at 58 °C on the Arrhenius plot. At temperatures lower than 58 °C, only the dissociation of the helical structure existed and the rate of relaxation from helix to worm-like conformation was negligible. At temperatures higher than 58 °C, the transition from helical chain to worm-like chain was the rate-determining step. The intrinsic fluorescence technique demonstrated its practicability in detecting kinetic processes of sPS/chloroform gel in the course of melting.

## 1. Introduction

As a high stereoregularity polymer, syndiotactic polystyrene (sPS) has attracted long-standing interest because of its high melting point, fast crystallization rate, and good chemical stability [1]. The polymorphic behaviors related to different molecular conformations and different chain packing structures have been extensively studied over the years [2,3,4,5,6,7,8]. Two types of molten crystalline structures have been distinguished, described as *α* and *β* conformations. The conformation of the polymer chains is a planar zigzag. The melt-crystallized crystalline morphologies are complicated. There are two limited disordered variants, *α*′ and *β*′, and two limited ordered variants, *α*″ and *β*″ [2,3,4]. An Δ crystalline phase which includes two identical cavities can be produced in the presence of solvent, and a compound between polymer and solvent is constituted in Δ form [5,6,7,8]. Moreover, a highly-oriented guest relative to the polymer host can be achieved [9,10].

Syndiotactic polystyrene (sPS) was found to compose thermo-reversible gels in a number of solvents [11,12,13,14,15], and many studies about sPS gels have been reported since Kobayashi et al. [16]. Cooling an sPS solution at low temperature leads to transparent gels. The gels are physical gels formed through physical bonds instead of covalent ones. Here, the thermo-reversible gel consists of 21-helix conformation chains, in which solvent molecules are intercalated between sPS chains [17]. Paste-like opaque gels were produced when sPS solutions with bulky solvents such as octadecyl benzoate and 1-chlorotetradecane were cooled [18,19]. sPS chains are in the highly ordered all-trans TT skeletal conformation [20]. The gelation mechanism of real physical gels is not very clear because some systems are not real physical gels considering the criteria proposed by Guenet [13,21]. A few studies concerned with the melting process of real gels in benzene, toluene, and chloroform were reported with a neutron scattering technique [19,22,23]. However, the relevant kinetic study has not been conducted. Many methods have been applied to study the gelation process of sPS, such as small-angel neutron scattering (SANS), infrared spectroscopy (IR), differential scanning calorimetry (DSC), small and wide angle X-ray scattering (WAXS), scanning electronic microscopy (SEM), etc. [13,14,15,24]. Intrinsic fluorescence is an effective and nondestructive research tool for investigating the chemical and physical characteristics of macromolecules, and can also be used for monitoring changes of the microenvironment [25]. It has been directly applied to study the sol–gel transition of the iPS (isotactic polystyrene)/decalin system [26], but has not yet been used to obtain structural information of sPS systems during melting. Therefore, it is expected to be an effective tool to probe the evolution of the structural conformation of sPS chains and their aggregates [20].

Fluorescence anisotropy is a powerful technique for the analysis of molecular interactions, and was first described by Perrin [26]. The technique has been extensively used in biological and diagnostic systems, such as for the study of small molecules with proteins, antigen–antibody, hormone–receptor, and protein–DNA interactions [27,28,29,30]. Meanwhile, fluorescence anisotropy has been used to detect molecular orientation in solid polymer films, polymer melts, and fibers [31,32,33,34,35,36,37] by mixing small amount of dyes into the polymer matrix. Furthermore, fluorescence anisotropy has been applied in sPS and iPS gels to determine the free volume in thermo-reversible gels or in polymer host nanoporous films to monitor the orientation of guest molecules by incorporating sizeable dye molecules (e.g., naphthalene) into the polymer–solvent system or into sPS nanoporous films [20,38,39,40,41,42]. However, the relaxation behavior of sPS chains at various temperatures has not been considered using the fluorescence anisotropy technique.

In order to obtain specific mechanisms and dynamics of structural relaxation behavior of sPS gels, the present work investigates the progressive relaxation of chains in the gel melting process by intrinsic fluorescence and fluorescence anisotropy techniques.

## 2. Materials and Methods

Syndiotactic polystyrene (sPS) was provided by Dow Chemical (M_w_ = 230,000). The fluorescence probe molecule used in the polarization measurements was naphthalene (NP). sPS/CHCl_3_ solutions with or without NP were prepared by dissolving a desired amount of compound in a closed glass container at 80–90 °C. The gels were produced by quenching hot sPS/CHCl_3_ solutions at −20 °C. 

Fluorescence excitation spectra and fluorescence emission spectra were detected with a combined steady state and fluorescence lifetime fluorimeter (FL920) attached with an electronically-controlled programmable thermostat to control the temperature within ±0.1 °C. The illuminant was a 450 W xenon lamp. The excitation slit was 2.5 nm and the emission slit was 5 nm for the emission monochromator. In the experiments, the samples were heated from 25 to 65 °C at 0.5 °C/min.

For the fluorescence anisotropy experiments, sPS/CHCl_3_ gels were obtained by dissolving sPS and NP in CHCl_3_ at 80 °C and kept at −20 °C overnight before measurement. The concentrations of sPS and NP were 10 g/L and 0.02 g/L, respectively. All measurements were performed with an optical path length of 1 cm. The anisotropic values were obtained by measuring values for 100 s at 330 nm more than three times. The excitation wavelength for NP was 280 nm.

UV/Vis spectra were collected with a UV-3150 spectrophotometer (Shimadzu Corporation, Kyoto, Japan).

Scanning electron microscopy (SEM) was performed to observe surface structures of the sPS/CHCl_3_ gels using a ZEISS SUPRA 55. sPS/CHCl_3_ gels were deposited at 25, 55 °C and after melting respectively and dried 48 h. The samples were coated with a quite thin layer of Platinum before measurements.

## 3. Results and Discussion

### 3.1. Intrinsic Fluorescence Spectra of sPS/CHCl_3_ Solution and Gel

**Figure 1:**
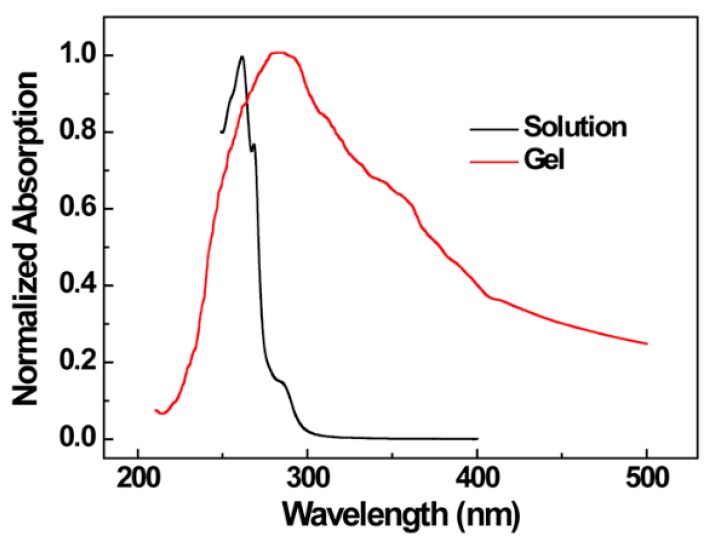
Normalized absorption spectra of 4 g/L syndiotactic polystyrene (sPS)/chloroform solution and gel.

In Figure 1 the absorption spectra of 4 g/L sPS/chloroform solution and gel at 25 °C are shown. It can be seen that a prominent absorption peak for sPS/chloroform solution was present at 260 nm and was accompanied by another weak peak at 269 nm. The absorption decreased over 270 nm and then reached 0 over 300 nm. However, there was a maximum absorption peak at about 280 nm for sPS/chloroform gel. Meanwhile, it can be seen that the absorption spectrum of the gel was much broader than that of the sPS/chloroform solution, which is attributed to the slightly hazy of the gel. This haziness also means that the excited state of the chromophore was destabilized by molecular aggregation and arrangement [43]. The excitation wavelength (260 nm) in the emission spectra could be obtained from the absorption spectra of the sPS solution and gel.

**Figure 2:**
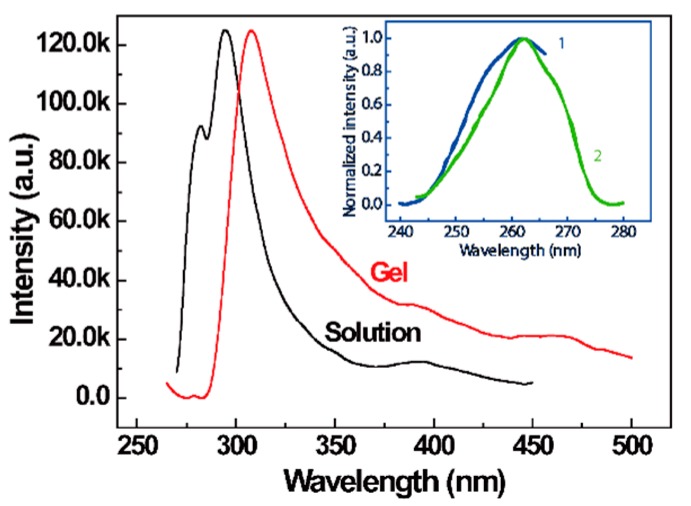
Emission spectra of sPS solution and gel (C = 4 g/L) excited at 260 nm. The inset gives the normalized excitation spectra. Curves 1 and 2: excitation spectra of gel, emission at 280 and 320 nm, respectively.

Figure 2 shows the emission spectra of sPS solution and gel (excitation at 260 nm) and the excitation spectra of sPS gel (emission at 280 and 320 nm). As exhibited in Figure 2, two fluorescence peaks appeared at 280 nm and 295 nm in sPS/chloroform solution. However, only one peak appeared at about 308 nm in the gel. Here, the emission at 280 nm is attributed to monomer emission [44]. Both 295 and 308 nm peaks belong to excimer emission because sPS presents a broad excimer from 290–350 nm centered at 315 nm [45]. The appearance of the excimer at high concentration is ascribed to intra-chain or inter-chain interactions among the phenyl moieties [46,47]. The red shift of the emission spectra of sPS gel compared with solution comes from the fact that different aggregate state of polymer chains or chromophore aggregates existed in solution and gel. The inset in Figure 2 gives the fluorescence excitation spectra achieved at 280 nm and at 308 nm. Both of them produced a maximum peak at around 260 nm, which means that the fluorescence emission peak at 280 nm and 308 nm came from the same chromophore. Meanwhile, the results also support the fact that the excimer formation of phenyl groups can occur in sPS chains. A similar phenomenon can be observed in the excitation spectra of pyrene/cyclodextrin solutions [48].

### 3.2. Intrinsic Fluorescence Spectra of sPS/CHCl_3_ Gel upon Controlled Heating

Thermal effects have appreciable impact on the functional characteristics of sPS gels. However, the mechanism of the effect of heat on sPS gel has not yet been fully studied. The present work is focused on revealing the evolution of polymer chains during melting using the intrinsic fluorescence method. Research is focused on the evolution of molecular conformation and their realignment when sPS/chloroform gel is heated. Quantitative examinations of the isothermal melting dynamics of sPS/chloroform gel were also performed.

**Figure 3:**
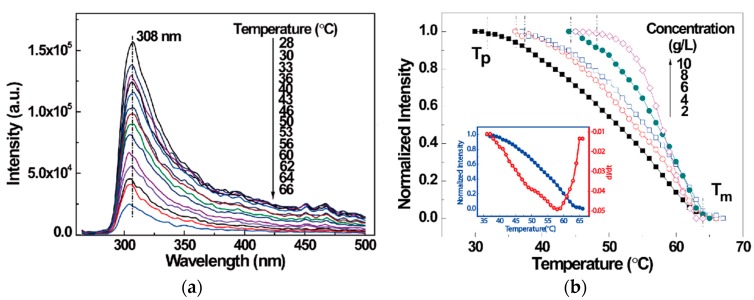
(**a**) Temperature-dependent intrinsic fluorescence spectra for syndiotactic polystyrene (sPS)/chloroform gel (C = 4 g/L); (**b**) Temperature dependence of normalized intrinsic fluorescence intensities at 308 nm for sPS/chloroform gels at various concentrations: 2, 4, 6, 8, and 10 g/L, respectively. The inset gives the temperature dependence of intrinsic fluorescence intensity for sPS/chloroform gel (C = 4 g/L) and the differential curve of I_308_ against temperature.

Figure 3a shows emission spectra for the sPS/chloroform gel (C = 4 g/L) obtained upon controlled heating. The emission intensity decreased with increasing temperature. The reduction of I_308_ can be attributed to the fact that the gels with oriented helical rods transformed into a homogeneous solution with worm-like conformation [19]. In this case, the interaction between solvent and aromatic rings gradually decreased during heating. In order to investigate the gel melting process, temperature dependence I_308_ curves for various concentration sPS/chloroform gels are provided in Figure 3b. It is clear that I_308_ is nearly constant during the initial period of heating, and then presented a notable reduction in the higher temperature period, corresponding to a significant variation in the gel phase [19]. Hence, onset temperatures of structural relaxation behavior, T_p_, could be determined as 32, 37, 39, 45, and 49 °C for 2, 4, 6, 8, and 10 g/L gels, respectively. T_p_ increased with increasing concentration, because more junction points and helical structures can be produced in the gel when the sPS concentration is high, which restrains the movement of molecular chains [25]. Eventually, I_308_ reached a plateau, suggesting that the relaxation behavior entered the last period. From Figure 3b, the melt temperatures of sPS with various concentrations could also be obtained. It can be seen that the melt temperature (*T*_m_) was almost the same (*T*_m_ = 64 °C). This result is lower than that determined by DSC technique [14]. The inset in Figure 3b gives temperature derivative of I_308_ for the 4 g/L sPS/chloroform gel. The derivative curve represents the structural relaxation rate. Thus, the structural relaxation behavior first accelerated and then decelerated with increasing temperature, obtaining the fastest rate at about 58 °C. Therefore, the intrinsic fluorescence intensity at 308 nm reflects the feasibility of this technique for the progressive relaxation behavior of sPS/chloroform gels.

### 3.3. Fluorescence Polarization of NP in sPS/Chloroform Gel upon Controlled Heating

The fluorescence anisotropy is independent of the intensity of the light and the concentration of the chromophore. Generally, when a chromophore is excited by polarized light, the emission is polarized when (i) the chromophore molecular motion is very slow or binding with large molecules [28] and (ii) energy migration and/or energy transfer do not occur. When excitation energy can hop among molecules or chromophore motion is fast enough, the anisotropy of the emission is equal to zero. Thus, information about molecular motion and/or energy transportation can be obtained by means of emission anisotropy.

Based on the mechanism of fluorescence polarization discussed above, naphthalene (NP) molecules can be adopted in the sPS/CHCl_3_ system to investigate the variation of chain conformations in terms of interaction between polymer and naphthalene molecules. The applicability of NP in sPS gels when applying fluorescence polarization has already been demonstrated by Itagaki and Guerra groups [20,38,39]. For sPS/CHCl_3_ gels, there are two possible locations of solvent molecules in the gel: one is the area (defined as region I) where solvent molecules gather; in this area, solvent molecules cannot interact with sPS chains. The other is an area where sPS chains associate together (region II); in this area, compounds consist of polymer chains and solvent molecular here, solvent molecules stay among sPS helical chains. Thus, it can be concluded that the anisotropy value of the mixture contains two emitting species with anisotropy values *r_i_* indicating the anisotropies of the individual species and fractional intensities if located in these two regions of the gel. The anisotropy is given by:

(1) $$\bar{r}=\sum_{i}f_{i}r_{i}$$

In region I, the NP molecules are not constrained as in fluid solution, and *r* = 0. Because the place accessible for NP in region II is less than 0.2%, all of the NP in the sPS gel remain in region II [20]. The phenomenon showsthat the anisotropy comes from the NP molecules in region II. Therefore, the anisotropy variation represents the motion of the chromophore molecules in region II and thus reflects the conformation change in polymer chains.

The steady-state fluorescence anisotropy can be expressed by applying the Perrin equation:

(2) $$r=\frac{r_{0}}{1+\tau /\phi }$$

where *r*_0_ is the maximal fluorescence anisotropy value lacking any rotational motion, *τ* is the fluorescence lifetime, and *Φ* represents the rotational relaxation time. *τ/Φ* does not vary much with temperature due to the similar temperature dependencies of *τ* and *Φ* even at the phase transition process [46]. Therefore, the variation of fluorescence anisotropy can express the conformation relaxation behavior in polymer chains at various temperatures.

**Figure 4:**
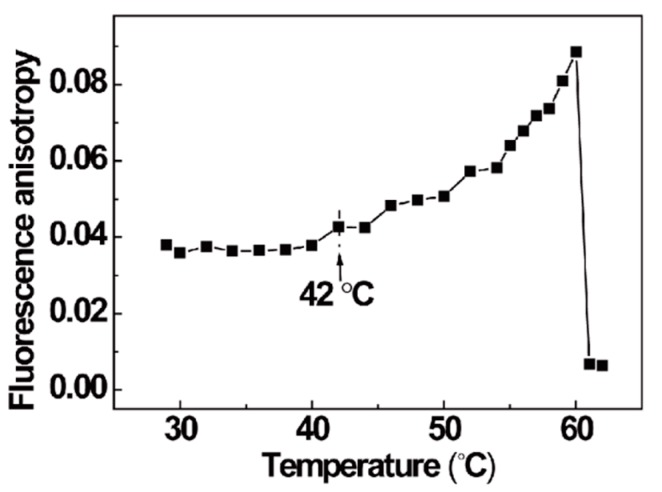
Fluorescence anisotropy as a function of temperature. The heating rate was 0.2 °C/min.

Figure 4 shows the temperature dependence of fluorescence anisotropy in the sPS/CHCl_3_ system upon controlled heating. It can be observed that the anisotropy value was almost constant before 42 °C. The smaller *r* values of 0.04 obtained below 42 °C are consistent with the values in Δ phase of sPS [40]. The value was smaller than the corresponding value in amorphous phase (0.07), and there are two possible reasons for this: (i) larger molecular motion of the NP molecules appears in the clathrate of the Δ phase; (ii) more efficient energy migration among NP molecules in the clathrate of the Δ phase. Previous study has shown that the mobility of chromophore molecules is low as guest of the Δ phase, but it is larger when the chromophore molecules were absorbed in the amorphous phase [47]. Thus, the smaller anisotropy values in sPS/CHCl_3_ gel indicated that the energy migration was dominating. Therefore, at low temperature, the helical polymer chain conformations in Δ phase contribute to excitation energy transformationdue to the short distance of the NP in the clathrate. The fluorescence anisotropy value presented a sharp increase in the temperature range of 42–60 °C. When the temperature was higher than 42 °C, the evolution of the sPS chain conformation made the anisotropy value begin to increase. The increase of anisotropy meant that the efficiency of singlet energy migration reduced gradually due to the increasing distance between NP molecules from 42–60 °C. In this process, it can be demonstrated that the triple helical aggregates presented in the sPS gel at low temperature dissociated into single helixes gradually as temperature increased. Meanwhile, some NP molecules could separate from the clathrate, but the contribution is negligible. According to the previous study, the anisotropy value for NP absorbed in amorphous sPS films was 0.07. However, in our sPS gel system, when the temperature was at 60 °C, the anisotropy value reached 0.09, which is much higher than 0.07. This verifies that the distance of NP molecules in separated helical chain conformation is larger than that in coil conformation. Eventually, the anisotropy value decreased to 0.06 above 60 °C. The reduction of anisotropy illustrates that the contribution of NP molecules mobility to anisotropy exceeded that of energy migration between NP molecules. This originated from the decrease of the binding interaction between NP and the polymer chain due to the relaxation behavior of the polymer chain from helical to coil conformation and the quick diffusion of NP molecules into region I at high temperature. Meanwhile, it can be seen that the fluorescence anisotropy was about 0.06. Thus, it can be concluded that there were still some helical conformations binding with NP molecules in the melting sPS/CHCl_3_ system.

**Figure 5:**
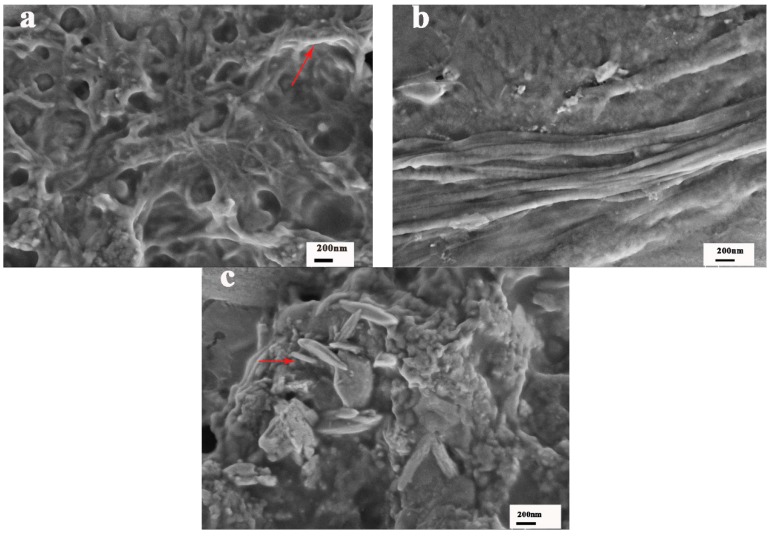
SEM images of the (**a**) helical network structure (indicated with red arrow) in the sPS/chloroform gel at 25 °C have been highlighted; (**b**) Dissociated helical structure at 55 °C; (**c**) Worm-like structure after melting (indicated with red arrow).

The helical structures, dissociated helical structures, and wormlike structures at different periods were further observed by SEM. It was found that wheat-like helical aggregates existed at room temperature (Figure 5a). An SEM image obtained at 55 °C is visible in Figure 5b. It is observed that dissociated helical structures were present. The images in Figure 5c provide the detailed structures after gel melting. Here, wormlike structures with short helical segments are visible.

**Figure 6:**
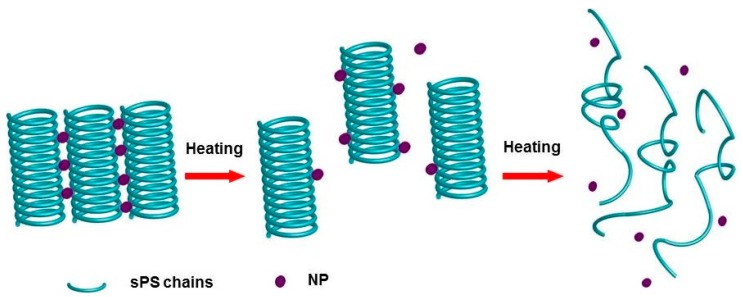
Schematic representation of the conformation change of sPS chains and positions of naphthalene (NP) molecules in the sPS/CHCl_3_ system upon controlled heating.

Following the analysis above, the gel melting process during heating can be stated as a dissociation process of helical aggregates at low temperature—in this case, NP molecules still combined with helical chains. At higher temperature, an incomplete conformation relaxation behavior from helix to coil conformation took place, and almost all of the NP molecules separated from the polymer chains. The results agree with those obtained by the resonance light scattering method [48]. A schematic representation of the structural evolution of the sPS chain upon controlled heating is shown in Figure 6.

### 3.4. Isothermal Melting Dynamics of sPS Gels upon Controlled Heating

**Figure 7:**
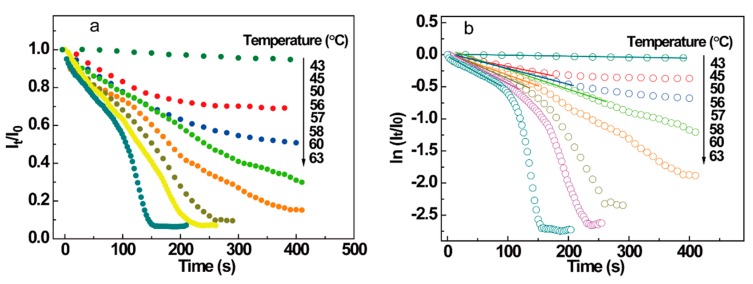
(**a**) $\frac{I_{t}}{I_{0}}$ vs. time plot and (**b**) $\ln\frac{I_{t}}{I_{0}}$ vs. time plot for 6 g/L sPS/chloroform gel at different temperatures.

The above sections show the feasibility of fluorescence and anisotropy in detecting microstructural development and polymer chain motion in non-isothermal heating conditions. The transformation of chain structure under isothermal conditions can also be detected by the intrinsic fluorescence technique. To give insight into the structural variation during the melting of an sPS gel, normalized emission intensity is presented with melting time in Figure 7. The normalization procedure was applied to the intensities to cancel small differences in the samples, facilitating a quantitative comparison between the intensities at different temperatures.

By analyzing the data in Figure 7, the structural change in the isothermal melting process of sPS/CHCl_3_ gel (e.g., when C = 6 g/L) can be described as follows. When the temperature was below 58 °C, the intensity decreased with time slowly and finally remained invariant. Above 58 °C, the intensity decreased with time in the initial stage, then the intensity decreased significantly and the intensity finally remained constant. The apparent two parts in the intensity curve indicate that the melting process is complicated. To express the processes clearly, Figure 7b shows the $\ln\frac{I_{t}}{I_{0}}$ vs. time plots for a C = 6 g/L sPS/chloroform gel at different temperatures. The plots were nearly linear when the temperature was below 56 °C, and the period is attributed to the dissociation of the sPS gel from a helical structure to individual helixes. The plot changed obviously as temperature increased. At 57 °C, good linearity was seen in the primary period of the $\ln\frac{I_{t}}{I_{0}}$ vs. time plot, and the line deviated from linearity as time increased. The deviation from the linear portion indicates that another transformation process from rigid helical structure to worm-like chain conformation may have been accompanied by the relaxation behavior from the helical network to individual helixes. The deviation became more severe when the temperature exceeded 58 °C, clearly demonstrating that two relaxation behaviors co-existed. Further, it is expected that the relaxation from helical network to individual helixes dominated the processes at low temperature and the relaxation from individual helixes to worm-like chain structure gradually became more dominant as temperature increased.

The apparent rate constants of chain conformation transformation (*k*) could be acquired from the slope of the linear part in Figure 7b, and they are presented in Table 1. In the current research, the dynamics in the linear period is investigated. The relationship between the apparent rate constants and the temperature of the reaction can be provided by the Arrhenius equation [49]:

(3) $$k=k_{0}\exp(\frac{E_{a}}{RT}),$$

where *k*_0_ is a frequency factor (s^−1^), *E_a_* is the melting activation energy (kJ mol^−1^), and *T* is the absolute temperature (K). For normal processes, the plot of *ln* (*k*) vs. the reciprocal of temperature is a straight line.

**Figure 8:**
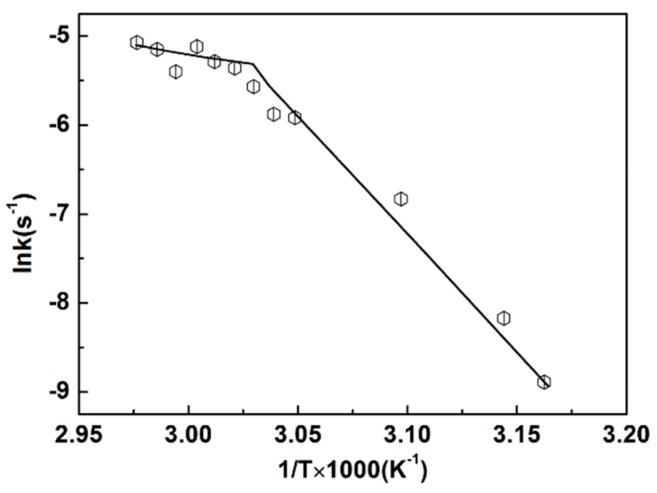
Arrhenius plot for 6 g/L sPS/chloroform in the gel melting process.

However, as shown in Figure 8, the apparent rate constant for the sPS/chloroform gel melting process was bi-phasic and it showed an obvious edge at 331 K (58 °C). The slope at low temperature (lower than 58 °C) was greater than that at higher temperatures (above 58 °C), confirming that the melting process is a two-step process involving the dissociation of the helical network structure at lower temperature and the relaxation behavior from individual helixes to a worm-like structure at higher temperature [48].

### 3.5. Thermodynamic Considerations of sPS Gels upon Controlled Heating

During the melting process, thermodynamic and kinetic activation parameters for the sPS gel could be obtained through intrinsic fluorescence. The *E_a_* values could be calculated from the Arrhenius equation in the two temperature ranges. The activation free energy (Δ*G*), the activation enthalpy (Δ*H*), and the activation entropy (Δ*S*) of sPS gels at different temperatures could be obtained with the Eyring equation (Equation (4)) during the thermal melting process [49].

(4) $$k_{}=(k_{B}^{}T/h)\exp(-\Delta G/RT),$$

and the relationships

(5) $$\Delta G=-RT\ln\frac{k\cdot h}{k_{B}\cdot T},$$

and

(6) $$\Delta H=E_{a}-RT,$$

(7) ΔG=ΔH−TΔS.

Then,

(8) $$\Delta S=\frac{\Delta H-\Delta G}{T},$$

where, *h* represents Planck’s constant and *k_B_* represents Boltzmann’s constant. The detailed Δ*H*, Δ*G*, and Δ*S* values are listed in Table 1.

The value of Δ*G* remained almost constant over the temperature range studied. It was 97 kJ·mol^−1^, which is characteristic of the dissociation course from helical network structure to individual helixes for sPS/chloroform gel in the heating process. Positive Δ*S* values obtained at low temperatures reflect the low orderly state corresponding to the dissociation course from a helical network structure to individual helixes. On the other hand, at higher temperatures, negative Δ*S* values could be gained. The difference between the Δ*S* values is attributed to the entropy change of single chains. Kauzmann and Eyring [50] pointed out that the whole segment acts as a unit in movement for a stiff polymer chain, and therefore the Δ*S* is positive. The coil structure can be considered as flexible polymer chains containing many segments per chain, and the Δ*S* is likely to become negative. In the present work, the sPS helical network structure was disrupted into separated helical chains due to the destruction of weak inter-chain bonds at low temperature and the Δ*S* was positive, while the relaxation from helical to worm-like chain was the dominant factor that led to a negative Δ*S*. Furthermore, from the values in Table 1, it is observed that the helical network structure dissociation and subsequent chain relaxation behavior provided completely different values. The biphasic Arrhenius plot represents that there were two distinct rate-determining steps for sPS melting rates [49,51]. It is well-known that the slowest process is the rate-determining step, and thus it is possible to infer the rate-determining step from the thermodynamic and kinetic values of this study, regardless of how many steps are involved. The high Δ*H*, *E_a_*, and positive Δ*S* values at the low temperature range reveal that the dissociation of the helical network structure was the rate-determining step. In this process, the aggregated structure of the helical conformations is disrupted into separated helical conformations. The low *E*_a_, Δ*H*, and the negative Δ*S* values in the high-temperature range represent an increase in order. Although the helical network was dissociated as well, the dissociation process was rapid compared with the relaxation process. The result suggests that the relaxation process from helical to coil conformer became the rate-determining step in this temperature range.

## 4. Conclusions

The evolution of sPS conformation and the isothermal melting dynamics were investigated using intrinsic fluorescence and fluorescence anisotropy techniques in the melting course of sPS/CHCl_3_ gels. Intrinsic fluorescence intensity correlated with the development of sPS conformation. The gel melting process is a dissociation process of the triple helical aggregates at low temperature, followed by an incomplete conformation relaxation behavior from helix to coil conformation. The results obtained by intrinsic fluorescence technique are in good agreement with the experimental results obtained by the RLS (resonance light scattering) spectroscopy technique. A sharp turning point was achieved on the Arrhenius plot, indicating that there are two steps in the gel melting process. The thermodynamic and kinetic parameters were acquired from Eyring and Arrhenius equations. Positive and negative Δ*S* values were achieved at low and high temperature, respectively. Three helical aggregates’ dissociation process is the only step below the transition temperature, while the relaxation from helix to coil becomes the rate-determining step above the transition temperature.

## Figures and Tables

**Table 1:** Thermodynamic and kinetic parameters for 6 g/L sPS/chloroform gel during the melting process.

*T* (°C)	*K* (×103 s^−1^)	*E_a_* (kJ·mol^−1^)	Δ*H* (kJ·mol^−1^)	Δ*G* (kJ·mol^−1^)	Δ*S* (kJ·mol^−1^)
43	0.13	141.94	139.31	101.09	0.12
45	0.28		139.29	99.66	0.12
50	1.08		139.25	97.66	0.13
55	2.69		139.21	96.73	0.13
56	2.80		139.20	96.92	0.13
57	3.82		139.19	96.38	0.13
58	4.71	23.44	20.69	96.10	−0.23
59	5.05		20.68	96.20	−0.23
60	5.99		20.67	96.03	−0.23
61	4.52		20.66	97.11	−0.23
62	5.80		20.65	96.71	−0.23
63	6.28		20.64	96.79	−0.23
